# Alcohol and nicotine: quitting both or keep smoking?

**DOI:** 10.1192/j.eurpsy.2025.150

**Published:** 2025-08-26

**Authors:** G. Dom

**Affiliations:** University of Antwerp, Antwerp, Belgium

## Abstract

**Abstract:**

people with alcohol- and substance use disorders have disproportionally high levels of tobacco use compared with the general populations. This concerns not only the prevalence of nicotine dependence but also the intensity of their smoking behaviour. Importantly, regarding the negative consequences the combined effects of alcohol and smoking are exponentially. Efforts to include smoking cessation treatment within the treatment programs for AUD and other SUD patients need to be intensified.

**Disclosure of Interest:**

None Declared

